# Ruptured Ovarian Artery Aneurysm in a Postmenopausal Female: Case Report

**DOI:** 10.5811/cpcem.1643

**Published:** 2024-03-25

**Authors:** Raj Patel, Amy Russell, Melanie M. Randall

**Affiliations:** Riverside University Health System, Department of Emergency Medicine, Moreno Valley, California

**Keywords:** *intra-abdominal hemorrhage*, *ovarian artery*, *aneurysm*, *case report*

## Abstract

**Introduction:**

Ovarian artery aneurysm is a rare diagnosis, primarily associated with late pregnancy and the postpartum period. It can cause life-threatening hemorrhage when ruptured. Even more rare are ovarian artery aneurysms in postmenopausal women.

**Case Report:**

We present a case of a postmenopausal female presenting to the emergency department with flank pain. Point-of-care ultrasound showed free fluid in the abdomen. She was diagnosed with an ovarian artery aneurysm on computed tomography angiography and treated successfully with embolization.

**Conclusion:**

Ruptured ovarian artery aneurysm is an uncommon cause of intra-abdominal hemorrhage in women.

CPC-EM Capsule What do we already know about this clinical entity?

*Ovarian artery aneurysms are rare and most often related to pregnancy. Rupture can lead to life-threatening hemorrhage.*

What makes this presentation of disease reportable?

*Few ovarian artery aneurysms have been described in postmenopausal women or presenting to the emergency department.*

What is the major learning point?

*Ovarian artery aneurysm is a rare but serious diagnosis that can present with significant intra-abdominal hemorrhage.*

How might this improve emergency medicine practice?

*This case adds to the existing differential diagnoses for intra-abdominal hemorrhage of unknown origin.*

## INTRODUCTION

Ovarian artery aneurysms (OAA) and pseudoaneurysms are primarily diagnosed during the late pregnancy and early postpartum periods. Hemodynamic and endocrine changes during pregnancy can lead to aneurysm formation.^1^ Other less common risk factors include trauma and chronic inflammatory conditions. The most common presenting symptom is significant abdominal and flank pain. Rupture of the aneurysm results in intraperitoneal and/or retroperitoneal hemorrhage often requiring emergent intervention. Incidence of this diagnosis is low.^2^ We present an uncommon case of a postmenopausal female found to have a ruptured left OAA.

## CASE REPORT

A 51-year-old, postmenopausal, gravida three, para three female presented to our emergency department (ED) with worsening left flank pain and new left-sided abdominal pain. She had been discharged from our hospital two days prior. During that admission, she underwent a left renal biopsy that was complicated by a perirenal and intraabdominal hematoma. Her medical history included systemic lupus erythematosus, chronic kidney disease, antiphospholipid syndrome, and hypertension. Her gynecologic history was significant for a prior cesarean section and a tubal ligation. She was on warfarin due to her hypercoagulable state.

**Image 1.**
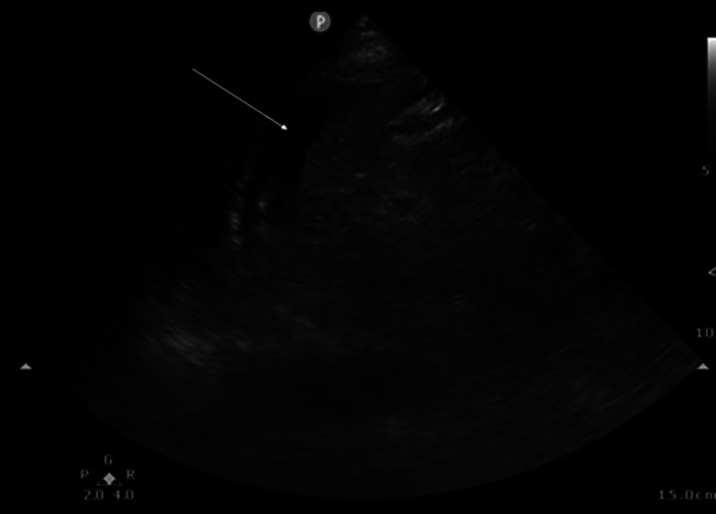
Point-of-care ultrasound showing free fluid in the left upper quadrant abdomen between the spleen and kidney (arrow).

Upon presentation to the ED, her vital signs were temperature 36.6° Celsius, heart rate 69 beats per minute, blood pressure 116/75 millimeters (mm) of mercury, respiratory rate 16 per minute, and oxygen level 96% on room air. Physical exam revealed a tired-looking female in mild distress. She exhibited left flank tenderness near her renal biopsy site with no external signs of bleeding or infection. Additionally, she had abdominal tenderness in the left upper quadrant (LUQ). There was no abdominal rigidity or signs of peritonitis. A point-of-care ultrasound was positive for free fluid in the LUQ (Image 1).

Her laboratory values were hemoglobin 6.6 grams per deciliter (g/dL) (reference range 12.1–15.1 g/dL), platelet count 374 × 10^9^ per liter (×10^9^/L) (150–450 × 10^9^/L), blood urea nitrogen 63 milligrams per dL (mg/dL) (7–30 mg/dL), creatinine 3.7 mg/dL (0.7–1.2 mg/dL), prothrombin time 15.8 seconds (10–13 seconds), and international normalized ratio 1.3 (0.89–1.16). The patient’s hemoglobin level three days prior was 9 g/dL.

**Image 2.**
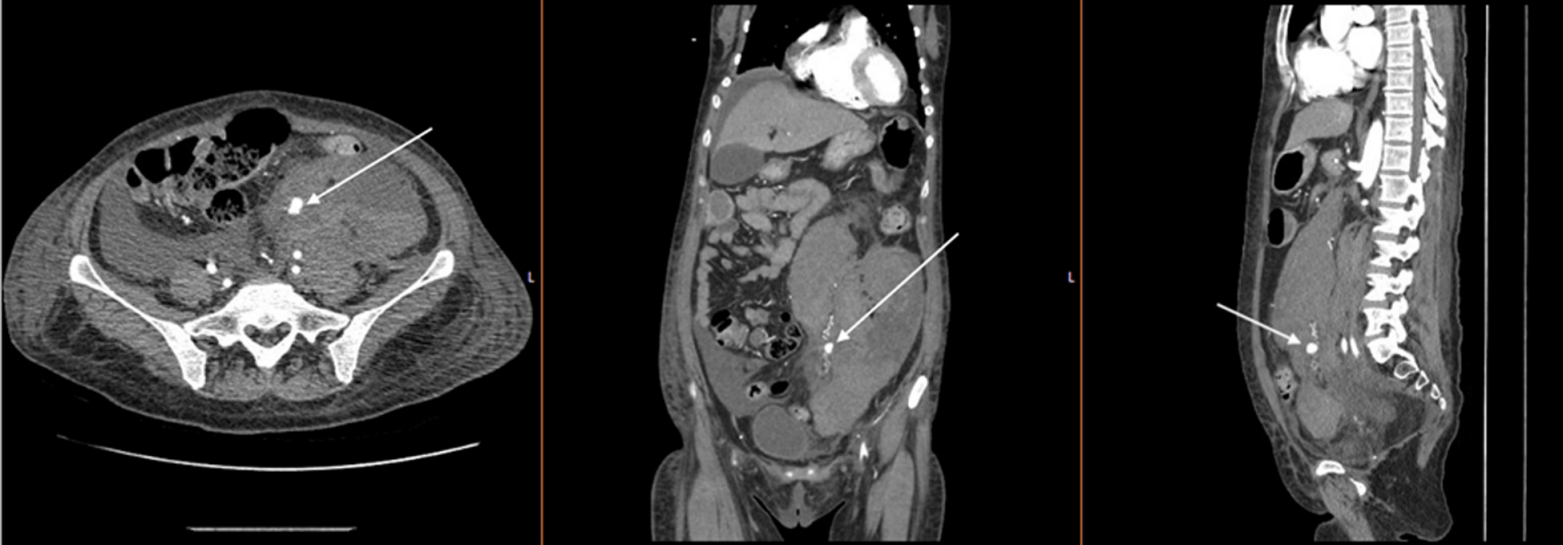
Computed tomography angiography demonstrating extravasation from left ovarian artery aneurysm (arrows).

**Image 3.**
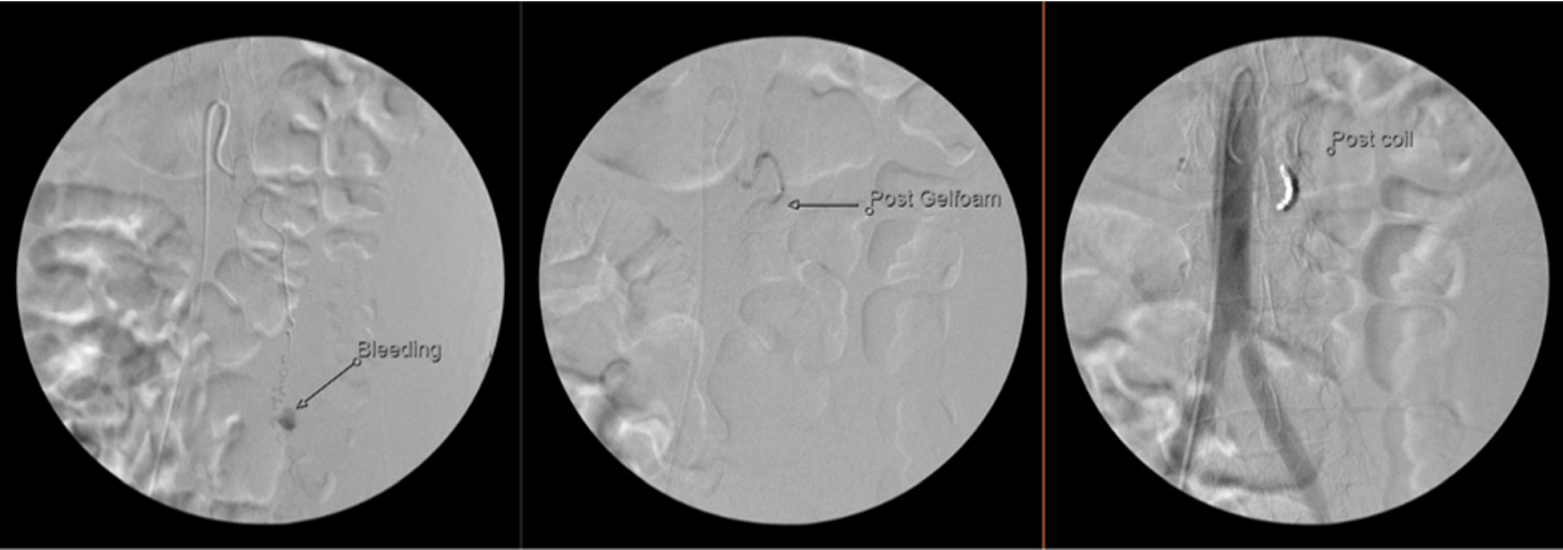
Angiography illustrating from left to right the ovarian artery aneurysm with active bleeding; after gelfoam embolization; and after coiling.

Interventional radiology (IR) was consulted due to the patient’s recent complicated biopsy, new laboratory derangements, and free fluid on ultrasound. Computed tomography (CT) angiography was recommended to assess for bleeding location and rule out abscess, perforation, or ischemia. Computed tomography of the abdomen and pelvis revealed a new 10 mm enhancement within the intra-abdominal hemorrhage. This abnormality was identified as a ruptured, bleeding distal OAA (Image 2). The patient was given one unit of packed red blood cells and taken to IR for definitive management. She underwent gelfoam and coil embolization of the left gonadal artery, resulting in cessation of bleeding (Image 3). After an uncomplicated hospital course, she was discharged.

## DISCUSSION

Ovarian artery aneurysm rupture is a rare but life-threatening diagnosis. The clinical presentation is non-specific but can include flank pain, abdominal pain, and hemodynamic instability. Imaging will show intraperitoneal and/or retroperitoneal fluid. Point-of-care ultrasound may assist in diagnosis if adequate intraperitoneal fluid volume is present. Definitive diagnosis requires CT angiography, traditional angiography, or direct operative visualization. Management requires IR embolization in a hemodynamically stable patient or laparotomy with ligation in an unstable patient.^2^^,^^3^

Although symptomatology and physical examination are non-specific, medical history may be helpful in the consideration of the diagnosis and the choice of imaging. While most commonly associated with pregnancy, OAA has also been described in patients with fibroids, chronic hypertension, trauma, vasculopathy, and in the postoperative phase.^2^^–^^11^ Increased plasma volume, increased cardiac output, and hormonal changes during pregnancy have been proposed as factors predisposing patients to OAA. Most documented cases occurred in multiparous women, suggesting that repeat pregnancy may be a risk factor due to the compounding effect of these physiologic changes.^2^^,^^4^

Finally, there are independent cases of OAA patients with chronic inflammatory conditions including chronic lymphocytic leukemia, rheumatoid arthritis, HIV, and lupus.^6^^,^^7^^,^^9^ The exact influence of chronic inflammation on aneurysm formation or growth is unclear.

## CONCLUSION

Ovarian artery aneurysm is a rare and serious disease most commonly associated with pregnancy. We present a rare presentation in a postmenopausal female in the ED. Due to its emergent nature, OAA can be considered in the differential diagnosis of a female patient presenting with nontraumatic abdominal or flank pain with certain appropriate risk factors.
